# Nutrition education: a questionnaire for assessment and teaching

**DOI:** 10.1186/1475-2891-4-2

**Published:** 2005-01-13

**Authors:** Mary Makowske, Richard D Feinman

**Affiliations:** 1 Department of Biochemistry. State University of New York Downstate Medical Center. Brooklyn, NY 11203 USA

## Abstract

It is generally recognized that there is a need for improved teaching of nutrition in medical schools and for increased education of the general population. A questionnaire, derived in part from a study of physician knowledge, was administered to first year medical students in order to assess their knowledge of various aspects of nutrition and metabolism, and as a teaching tool to transmit information about the subject. The performance of first year students was consistent with a generally educated population but there were surprising deficits in some fundamental areas of nutrition. Results of the questionnaire are informative about student knowledge, and immediate reinforcement from a questionnaire may provide a useful teaching tool. In addition, some of the subject matter can serve as a springboard for discussion of critical issues in nutrition such as obesity and markers for cardiovascular disease. A major barrier to improved teaching of nutrition is the lack of agreement on some of these critical issues and there are apparent inconsistencies in recommendations of government and health agencies. It seems reasonable that improved teaching should address the lack of knowledge of nutrition, rather than knowledge of official guidelines. Student awareness of factual information should be the primary goal.

## Background

Like many medical schools, SUNY Downstate Medical Center has been trying to improve the teaching of nutrition in the curriculum. The study presented here had two goals. First, we wanted to assess the knowledge of the first year class on the subject and, second, we hoped to use the questionnaire method, with immediate feedback, as a mechanism for imparting information on the areas covered. Whereas some form of nutrition education is part of the curriculum of most medical schools [1], it is generally believed that both medical professionals and the general public have serious limitations in their knowledge of the field [2,3]. Four of the 14 questions presented to the first year medical class were included in a recent survey of physicians published in this journal [4]. That paper was critical of the level of physician knowledge regarding nutrition. Therefore it seemed appropriate to compare performance of students with that of practitioners. In addition, the mechanism of providing information by presenting an anonymous quiz with immediate feedback could address one of the problems cited as a barrier to introducing nutrition into the medical curriculum – that is, that the first year basic science curriculum is already very concentrated, leaving little room for new material. The quiz format provides motivation and, because it is anonymous, does so in a relatively unstressful way.

Perhaps the most important problem in introducing nutrition into the medical curriculum is the lack of agreement on what should be taught. There is strong, even contentious debate about the most fundamental issues such as obesity, cardiovascular disease, and diabetes. Thus, concerns about inadequate physician knowledge frequently refer to ability to counsel patients according to standard guidelines [4-6]. Here we suggest that a more appropriate goal would be the understanding of basic nutritional information and that this information is not always in agreement with official guidelines. The details of these problems are left to Results and Discussion since we first want to offer the reader a chance to take the questionnaire as given to students.

The quiz is presented twice: first, as given to medical students, and then, a second time, with the answers and the comments given to students immediately upon completion of the quiz. A follow-up email that was sent to students after results were tabulated has also been reproduced. Results and Discussion provides data on student performance and additional discussion.

## Methods

The following is a verbatim reproduction (with addition of references) of the questionnaire given to first year medical students at SUNY Downstate Medical Center. The first four questions were taken from Flynn, *et al*. [4]. Following Flynn, we did not attempt to define the term "low fat diet" which is usually used without elaboration. No specific time limit was given for the quiz but a class of 111 students finished in about 20 min with a few stragglers. Students were given the answer sheet (shown after the quiz itself) in exchange for their questionnaire. Students were in the middle of the metabolism course, part of a subdivision characterized as Gastrointestinal Block. They had been taught bioenergetics and carbohydrate and lipid metabolism, and would be expected to know the answers to some of the questions based on that material. Other questions tested general nutrition knowledge while questions 12–14 emphasized material taught later in the course.

### Questionnaire

This questionnaire is anonymous and does not affect your grade. Make up a User Name (in case there is a follow-up) that only you know and that is not common, like Sherlock-37)

User Name _________________________________________________

The questionnaire is designed to test your general knowledge, not anything you have learned in the course.

1. A good source of monounsaturated fat is: (check all that apply)

_____ Butter

_____ Canola Oil

_____ Corn Oil

_____ Flaxseed Oil

_____ Olive Oil

_____ Safflower Oil

_____ Soybean Oil

_____ don't know

2. The diet component that is most likely to raise triglycerides is (select one)

_____ Fat

_____ Carbohydrate

_____ Protein

_____ don't know

3. In general, what effect does a low-fat diet have on triglycerides?

_____ Increase

_____ Decrease

_____ no change

_____ don't know

4. In general, what effect does a low-fat diet have on HDL-c (high density lipoprotein-cholesterol) ?

_____ Increase

_____ Decrease

_____ No change

_____ Don't know

5. In the past thirty years, the per cent fat in the American Diet has:

_____ Increased

_____ Decreased

_____ Stayed about the same.

6. The most energy dense food (most calories/gram) is:

_____ Carbohydrate.

_____ Protein.

_____ Fat.

7. High total blood cholesterol can be lowered significantly by:

_____ Diet

_____ Drugs such as statins

_____ Diet or drugs are equally effective

_____ Neither

8. The dietary change that is most likely to **increase **the risk of cardiovascular disease:

_____ unsaturated fat → saturated fat (that is, replace unsaturated fat with saturated fat)

_____ unsaturated fat → carbohydrate

_____ carbohydrate → unsaturated fat

_____ carbohydrate → saturated fat

_____ saturated fat → carbohydrate

_____ saturated fat → unsaturated fat

9. Glycemic Index measures the increase in blood sugar over 2 hours per gram of carbohydrate ingested, compared to glucose (=100). For each food indicate the approximate glycemic index as: H, high (70–100), M, Medium (40–70) or L, Low (< 40). You may enter a number if you think you know or can figure it out:

_____ white bread

_____ whole wheat bread

_____ ice cream

_____ carrots

_____ sucrose (table sugar)

_____ fructose

_____ bran muffin

_____ banana

10. The substances in the following list that either are themselves or are considered to contain large amounts of complex carbohydrates.

_____ white bread

_____ whole wheat bread

_____ ice cream

_____ fructose

_____ sucrose (table sugar)

_____ corn starch

_____ fiber

11. In the first list check the vitamins that are generally considered to have antioxidant activity. In the second list check the vitamins that are precursors for oxidative coenzymes. (check all that apply)

ANTIOXIDANTS

_____ ascorbic acid (vitamin C)

_____ niacin

_____ riboflavin

_____ thiamine

_____ pyridoxal phosphate (Vitamin B_6_)

_____ vitamin B_12_

_____ vitamin D

_____ vitamin E

REDOX PRECURSORS

_____ ascorbic acid

_____ niacin

_____ riboflavin

_____ thiamine

_____ pyridoxal phosphate

_____ vitamin B_12_

_____ vitamin D

_____ vitamin E

12. Megaloblastic anemia is a prominent feature of deficiencies of:

_____ Vitamin B_12_

_____ Folic Acid

_____ Neither

_____ Deficiencies of either

13. Addition of folic acid to the diet can relieve all the symptoms due to deficiencies of:

_____ Vitamin B_12_

_____ Folic Acid

_____ Both

_____ Neither

14. Vitamin B_12 _deficiency is most commonly seen in:

_____ children due to poor nutrition.

_____ children due to poor absorption.

_____ the elderly due to poor nutrition.

_____ the elderly due to poor absorption.

### Answers and feedback

Answers and comment were given to students immediately in exchange for their questionnaires. Students were polled about the quiz verbally and by Email. There were few responders although all were positive and included comments that they might not have read the material in the answers had they not taken the quiz first. The Answer Sheet is reproduced verbatim below and it should be understood that it contains some comments that are more colloquial than would be included in a more formal document.

### Answer Sheet

The first four questions appeared in a recent paper (Flynn M, *et a*l. *Nutr J *2003, **2**:19) that reported the results of a questionnaire designed to assess the level of knowledge of physicians. The authors were critical of the responses; the paper was entitled "Inadequate physician knowledge of the effects of diet on blood lipids and lipoproteins." In particular, "Physicians showed a poor understanding of the effects of changing the relative intake of carbohydrates and fats on triglycerides and HDL."

The rationale for the study were guidelines recommended by the Third Report of the National Cholesterol Education Program Adult Treatment Program (ATP III, Circulation 2002, **106**:3143–3421). The ATP III, compared to previous version, advocates lowering triglycerides as a secondary target to lowering LDL. The major conclusions:

• Half of the physicians did not know that canola oil is a good source of monounsaturated fat; 26% did not know that olive oil is also.

• Ninety-three percent (84% of cardiologists vs. 96% of internists) did not know that a low-fat diet, in general, would increase blood triglycerides.

• Approximately three-quarters (70% of cardiologists vs. 77% of internists) did not know a low-fat diet would decrease HDL; almost half (45%) thought that a low-fat diet would not change HDL.

• About one-half (47%; 22% of cardiologists vs. 53% of internists) did not know carbohydrate was the diet component most likely to raise triglycerides.

1. A good source of monounsaturated fat is: (check all that apply)

_____ Butter

_ **X **_ Canola Oil

_____ Corn Oil

_____ Flaxseed Oil

_ **X **_ Olive Oil

_____ Safflower Oil

_____ Soybean Oil

_____ don't know

It is generally considered that monounsaturated fats are protective of cardiovascular disease (as in the Mediterranean Diet). The effect of monounsaturated fat explains the anomaly in the so called Seven Countries study [7] which launched low fat recommendations. The two countries among the highest consumers of fat had roughly the highest (Finland) and the lowest (Crete) incidence of cardiovascular disease. This finding immediately led to a change in recommendations to lower saturated fat, rather than fat across the board. The persistence of a recommendation to lower total fat is now controversial.

**Figure 1:**
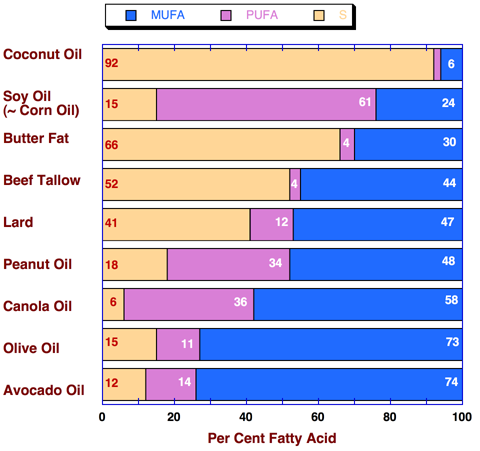
**Fatty acid composition of common dietary fats and oils. **Data from Figure 1.9 of reference [44].

Some people may be surprised when they actually look at the data (Figure 1). High concentrations of monounsaturated fats are found in olive oil (73 %) and canola oil (58 %), but the highest is in avocado oil, and there are fairly high levels in beef tallow and lard (44% and 47 %). Interestingly, beef tallow is what MacDonald's previously used to fry their French fries (which at the time got thumbs up from Julia Child) until pressured to switch to vegetable oil. Also of interest is that most of the saturated fat in beef fat itself is stearic acid which is not considered atherogenic [8].

You should also know that canola oil is named for CANadian OiL (there is no canola tree) and is a highly processed version of rapeseed oil (which *is *a plant in the Brassica family) and is the subject of popular controversy because of the possible production of *trans*-fatty acids during processing.

2. The diet component that is most likely to raise triglycerides is (select one)

_____ Fat

_ **X **_ Carbohydrate

_____ Protein

_____ don't know

3. In general, what effect does a low-fat diet have on triglycerides?

_ **X **_ Increase

_____ Decrease

_____ no change

_____ don't know

The phenomenon of carbohydrate-induced hypertriglyceridemia is well established in the literature [9-15]. It is the primary reason we ask patients to fast prior to having blood drawn for serum lipid analysis. It is also one of the arguments for low carbohydrate diets since the most predictable effect of such diets is a dramatic decrease in triglycerides compared with low fat diets [16-22]. Low carbohydrate diets also generally increase HDL (good) cholesterol while low fat diets lower HDL although this is somewhat less reliable than the triglyceride effect. An argument against low carbohydrate diets is that, whereas, on average, LDL frequently goes down, individual results are highly variable and LDL increases for some subjects [16-22].

4. In general, what effect does a low-fat diet have on HDL-c (high density lipoprotein-cholesterol)?

_____ Increase

_ **X **_ Decrease

_____ No change

_____ Don't know

5. In the past thirty years the per cent fat in the American Diet has:

_____ Increased

_ **X **_ Decreased

_____ Stayed about the same.

This is the major point of attack on current guidelines since the obesity epidemic correlates with a decreased percentage of fat in the diet (for men in some years, there was a decrease in total amount of fat) and increased carbohydrate consumption [23,24]. Defenders of current guidelines maintain it is simply that the wrong kind of carbohydrate (i.e., sugars and refined starches) is being consumed, and that affluence has increased the availability of food and portion sizes, and people are overeating "because it is there." On the other hand, published results of low carbohydrate diets show that they can be effective and therefore there is no great over-consumption even though portion sizes are unlimited. It is also argued that the wrong kind of fat (i.e., saturated and *trans *fats) is being consumed although no effect of fat *per se *on obesity, independent of calories, has been found.

6. The most energy dense food (most calories/gram) is:

_____ Carbohydrate.

_____ Protein.

_ **X **_ Fat.

The operational numbers in kcals/g are 4, 4, and 9 for carbohydrate, protein, and fat. This is the basis of traditional recommendations for low fat diets for obesity. The reduction in percentage of dietary fat and the obesity epidemic noted above, however, suggests that this is not a good universal principle. The role of macronutrient composition on satiety, taste, total consumption and effect on weight loss is largely unknown due to the multiple factors and individual differences, and some experimental support can be found for just about any idea.

7. High total blood cholesterol can be lowered most significantly by:

_____ Diet

_ **X **_ Drugs such as statins

_____ Don't know

_____ Diet or drugs are equally effective

_____ Neither

Drugs such as statins (HMGCoA reductase inhibitors) are very effective at reducing cholesterol. Diet can also be effective but usually less so. Combination diet and drugs, however, may be most effective but there is, again, not universal agreement as to what that diet should be.

8. The dietary change that is most likely to **increase **the risk of cardiovascular disease:

_____ unsaturated fat → saturated fat (that is, replace unsaturated fat with saturated)

_ **X **_ unsaturated fat → carbohydrate

_____ carbohydrate → unsaturated fat

_____ carbohydrate → saturated fat

_____ saturated fat → carbohydrate

_____ saturated fat → unsaturated fat

In addition to the effect on risk factors, epidemiologic evidence suggests that replacing fat with carbohydrate is deleterious. Replacing unsaturated fat with saturated fat will also increase the risk of cardiovascular disease [8,9,14,25]. Of course, in terms of obesity, reducing calories by removing fat and not replacing it with anything is good. Removing carbohydrate, however, may be better, at least in terms of cardiovascular risk as in references above.

9. Glycemic Index (GI) measures increase in blood sugar over 2 hrs per gram of carbohydrate, compared to glucose (=100). For each food indicate the approximate glycemic index as: H, high (60–100), M, Medium (40–60) or L, Low (< 40). You may enter a number if you think you know it:

**H (70) **white bread

**M (52) **whole wheat bread

**M (50) **ice cream

**M (47) **carrots

**H (70) **sucrose (table sugar)

**L (20) **fructose

**HM (60) **bran muffin

**M (50) **banana

The GI is a very rough indicator of rise in blood sugar and is influenced by absorption and the concentration of glucose. Fructose has a low GI (20) indicating slow conversion to glucose in 2 hrs but far from being considered a "good" sugar, at high levels may be very deleterious. The concept of glycemic load (GL) which corrects for the amount of carbohydrate per serving may be a better parameter but runs into problems about serving size. So muffins and candy bars have GL = 15 and carrots only 3 but 80 g of carrots may not be a lot for some people [26-28].

10. The substances in the following list that either are themselves or are considered to contain large amounts of complex carbohydrates.

_ **? **_ white bread

_ **? **_ whole wheat bread

_ **NO **ice cream

_ **NO **fructose

_ **NO **sucrose (Table Sugar)

_ **? **_ corn starch

_ **? **_ fiber

If you had trouble with this, it is because nobody knows how the term should be used and, in fact, we recommend it not be used at all. The original chemical definition – to some extent still used in organic chemistry – is that a complex carbohydrate is a polysaccharide (not mono- (glucose, fructose) or di- (sucrose, lactose)). Any starch, e.g. corn starch or white bread (poly-glucose: amylose or amylopectin) fits the definition, and it used to be nutritional dogma that, at least in terms of raising blood glucose, complex carbohydrates (that is, polysaccharides) were better than simple sugars. When the dogma was finally tested this turned out not to be true and the concept of glycemic index arose. It is clear from question 9 that white bread is nutritionally similar to pure glucose. Probably because of the evocative nature of the word, the term "complex" is still used. Sometimes it means foods that have a low glycemic index due to poor absorption usually due to the presence of fiber, but it is never precise. When people say complex carbohydrate they usually mean the carbohydrate recommended in their diet and missing from somebody else's diet. Suggested nomenclature is "polysaccharides, starch, high fiber," although fiber itself is a heterogeneous category.

11. In the first list check the vitamins that are generally considered to have antioxidant activity. In the second list check the vitamins that are precursors for oxidative coenzymes. (check all that apply)

ANTIOXIDANTS

_ **X **_ ascorbic acid (vitamin C)

_____ niacin

_____ riboflavin

_____ thiamine

_____ pyridoxal phosphate (Vitamin B_6_)

_____ vitamin B_12_

_____ vitamin D

_ **X **_ vitamin E

REDOX PRECURSORS

_____ ascorbic acid

_ **X **_ niacin

_ **X **_ riboflavin

_ **X **_ thiamine

_____ pyridoxal phosphate (Vitamin B_6_)

_____ vitamin B_12_

_____ vitamin D

_____ vitamin E

12. Megaloblastic anemia is a prominent feature of deficiencies of:

_____ Vitamin B_12_

_____ Folic Acid

_____ Neither

_ **X **_ Deficiencies of either

13. Addition of folic acid to the diet can relieve all the symptoms due to deficiencies of:

_____ Vitamin B_12_

_ **X **_ Folic Acid

_____ Both

_____ Neither

14. Vitamin B_12 _deficiency is most commonly seen in:

_____ children due to poor nutrition.

_____ children due to poor absorption.

_____ the elderly due to poor nutrition.

_ **X **_ the elderly due to poor absorption.

The most obvious symptom of a folic acid deficiency, anemia, is due to a requirement for folic acid in the synthesis of DNA. Deficiencies lead to poor maturation of red blood cells (megaloblasts). Megaloblastic anemia can also be caused by a B_12 _deficiency, which, indirectly, has the same effect.

There are only two reactions in humans requiring vitamin B_12_. First, vitamin B_12 _is a cofactor in formation of the amino acid methionine from homocysteine and the folic acid derivative, methyl-tetrahydrofolic acid (methyl-THF): 

Homocysteine + methyl-THF → Methionine + THF 

This explains the relation between dietary folic acid and high homocysteine which is a marker for cardiovascular disease and potential birth defects.

A deficiency in folic acid or the cofactor, B_12_, will prevent this reaction from occurring. The effect of B_12 _deficiency on folic acid is indirect: if methionine synthesis cannot be carried out, methyl-THF will build up ("methyl trap"). This is effectively a folic acid deficiency and anemia is the outcome. The second requirement for B_12 _involves organic acids and deficiencies can lead to neurologic damage. The anemia in a B_12 _deficiency may be successfully treated with folic acid, swamping out the methyl trap. Neurologic damage, however, may still occur unless the B_12 _deficiency is also treated. A dietary deficiency of B_12 _is rarely seen since little is needed and it is stored well. Deficiency is usually detected in the elderly due to decreased production of intrinsic factor, a protein required for absorption.

The considerations above bear on the recommendation to add folic acid to manufactured food. Critics point out that by preventing anemia, a B_12 _deficiency could be masked. Since the major deficiency is not dietary but absorptive, the problem can't be solved by simply adding B_12 _as well.

### Follow up

The following analysis was sent as an Email to students after the scores on the quiz were tabulated.

## Results of Nutrition Questionnaire

As indicated in the answer sheet, the first four questions are taken from a recent paper (Flynn, M., et al. (2003) *Nutrition Journal ***2**: 19) that reported the results of a questionnaire designed to assess the level of knowledge of physicians. Their conclusion was that "Physicians showed a poor understanding of the effects of changing the relative intake of carbohydrates and fats on triglycerides and HDL."

Interestingly the rationale for the study was determining "Physicians' ability to effectively counsel patients with elevated cholesterol to initiate a Therapeutic Lifestyle Changes Diet (TLC)" (as proposed by the Third Report of the National Cholesterol Education Program Adult Treatment Program (ATP III) which recommends lowering triglycerides as a secondary target to lowering LDL. The TLC Diet recommends total fat as 25–35 % and carbohydrate at 50–60 %. Paradoxically, Flynn, et al.'s assessment of physician knowledge focused on the deleterious effect of carbohydrate on triglycerides. Given this association, it is puzzling that ATP III would council people trying to lower triglycerides to undertake such a diet.

**Figure 2:**
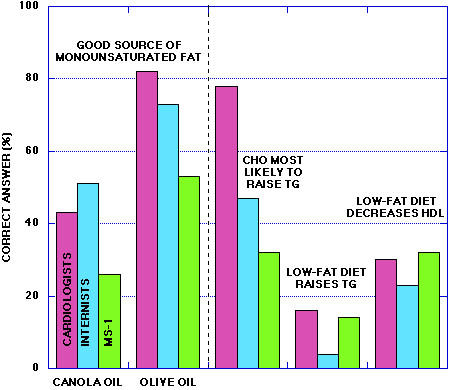
**Performance of physicians and first year medical students on questionnaire. **Data on physicians from reference [4].

In any case, your performance compared to their sample is shown in Figure 2.

Two questions of importance:

Question 5:

In the past thirty years, the per cent fat in the American diet has declined by about 10 %, close to the target level of 30% of total calories set back in the 70's. In order to explain why this has been associated with an obesity epidemic, many have blamed portion-size, the fast-food industry and consumers themselves. In any case, most people did not know that percent fat consumption had gone down.

Answers were:

Increased: 76 %

Decreased 22 %

Same 3 %

Question 6. Again, it was surprising that so many people did not know the relative energy density. The answers:

Carbohydrate 19 %

Protein 7 %

Fat 74 %

### Analysis

Questionnaires were collected from 111 students, all but 6 of whom answered all the questions. Student answers were tabulated and discrimination coefficients were determined by point biserial correlations [29] using LXR-TEST™ software (Logic eXtension Resources ). The discrimination coefficient varies between -1 and +1 and measures the extent to which performance on a particular question reflects performance on the quiz as a whole, that is, whether a question discriminates high performers from low performers. A typically high value of +0.4 means that the question was answered correctly by most students who did well on the exam and answered incorrectly by most who did not.

Because the questions had different goals (knowledge assessment vs. motivation for accepting new information), and because some (e.g. questions 2–4) are interrelated, no measure of performance or statistics were carried out for the quiz as a whole.

## Results and Discussion

### Student and physician knowledge of nutrition

#### Questions 1–8

Results from the first eight questions and the data from Flynn, et al. [4] are shown in Tables 1 and 2 and in Figure 2. Flynn, *et al*. used four questions that succinctly identified both practical and conceptual knowledge bearing on the ability to implement dietary recommendations from the Adult Training Program (ATP III) of the National Cholesterol Education Program (NCEP)[6].

**Table 1:** Student and physician responses (%)

	**Cardiologists**	**Internists**	**Students**	**discrim coeff**
**1. A good source of monounsaturated fat**				
Butter	4	4	8	0.39
**Canola Oil**	**43**	**51**	**26**	**0.21**
Corn Oil	13	16	22	0.32
Flaxseed Oil	12	10	25	0.32
**Olive Oil**	**82**	**73**	**58**	**0.35**
Safflower Oil	24	32	25	0.26
Soybean Oil	18	16	38	0.35
don't know	6	6	16	
				

**2. Diet component most likely to raise triglycerides**				
Fat	16	47	63	-0.2
**Carbohydrate**	**78**	**47**	**32**	**0.17**
Protein	0.8	0.6	0	
don't know	5	5	2	
				

**3. Effect of low-fat diet on triglycerides**				
**Increase**	**16**	**4**	**14**	**0.22**
Decrease	52	73	68	-0.17
no change	26	26	15	0.01
don't know	6	4	3	
				

**4. Effect of low-fat diet on HDL-c**				
Increase	11	24	31	-0.08
**Decrease**	**30**	**23**	**32**	**0.25**
no change	52	44	23	-0.18
don't know	7	9	14	

Discrimination coefficients indicate discrimination of high and low performers on quiz overall as described in the text. (correct answers in bold).

**Table 2:** Student Responses (%)

	**Students**	**discrim coeff**
**5. Past thirty years, per cent fat in American diet**		
Increase	76	-0.10
**Decrease**	**22**	**0.14**
Same	3	-0.10
		

**6. Most energy dense food**		
Carbohydrate	19	-0.15
Protein	7	-0.10
**Fat**	**74**	**0.19**
		

**7. High blood cholesterol lowered significantly by**		
Diet	14	0.06
**Drugs such as statins**	**21**	**0.17**
Diet or drugs equal	61	-0.15
Neither	0	
Don't know	0	
		

**8. Most likely to increase risk of CVD**		
UF t>gF	46	0.02
**UF -> CHO**	**7**	**0.11**
CHO -> UF	3	-0.27
CHO -> SF	23	0.09
SF -> CHO	2	0.00
SF -> UF	12	-0.08

First year students did not do as well as physicians at identifying sources of monounsaturated fats. On the other hand, the good discrimination coefficient indicates that knowledge of fat composition is a good indicator of overall knowledge (at least as assessed by general performance on this quiz).

Although a substantial fraction of cardiologists polled by Flynn knew that carbohydrate raised triglycerides (Figure 2), most internists and most medical students did not. Likewise, a very small fraction of first year students or physicians were aware of the association between low fat diets and two markers of CVD, triglycerides and HDL-c. As discussed in the student answers (see Methods), there is some irony in that the questions chosen by Flynn bring out the unfavorable effect of carbohydrate on triglycerides while the ATP III recommendation is to maintain 50 % carbohydrate in the diet. Student responses attest to the success of continued popular and government recommendations favoring low fat diets but the content of the answers raises the question of whether sufficient information is being disseminated. It further raises the question as to whether these recommendations, rather than the basic nutritional knowledge, should be communicated.

**Figure 3:**
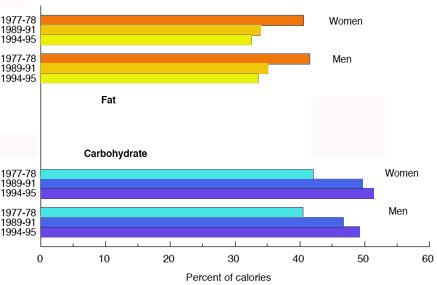
**Changes in fat and carbohydrate between 1977 and 1995. **Data from USDA as reported in reference [24].

Along the same lines, our questionnaire went beyond the area covered by Flynn to consider the changes in diet that have accompanied the epidemic of obesity. It has to be considered very surprising that only 22 % of an educated population knew that the per cent fat in the American diet has decreased (Figure 3); for men, in fact, the *total amount *of fat has decreased, whereas for women there has been a slight increase consistent with the much larger increase in caloric intake among women [24]. The observation of a decrease in fat and an increase in carbohydrate in parallel with the obesity epidemic remains as a serious challenge to traditional dietary recommendations. The reduction in fat in the diet from the 1970s to 1995 has been noted by one author [24] to provide a benefit in reduction in serum cholesterol from 213 to 205 mg/dL! Although more difficult to quantify, a decline in exercise is also a likely contributor to the epidemic, but it seems inappropriate, without further evidence, to ignore the *prima facie *evidence of the effect of macronutrients. Despite the clear correlation between higher carbohydrate, lower fat and obesity, government and health agencies rarely question the appropriateness of the original guidelines and have continued to recommend still higher carbohydrate and still lower fat [6,30,31]. Such recommendations have to be considered controversial and likely to change. For this reason we feel that one of the points brought out by our quiz and Flynn's is that nutritional facts rather than official recommendations should be the goal of nutrition education.

#### Question 6

We were surprised by some lapses in student knowledge revealed by the questionnaire. The most basic question – which macronutrient is the most energy dense – had only 74 % correct answers. To understand how surprising a response this is, it should be understood that the mean score on exams in this section of the medical course is typically 80 % and, on most exams, several questions are answered correctly by 98–100 % of the class. The National Board of Medical Examiners assumes knowledge of caloric value of macronutrients, and we had expected that it was common knowledge. The questionnaire result indicates that no fact in nutrition is too basic to be excluded from course material.

#### Questions 7–8 – Diet and Cholesterol

An overwhelming number of medical students believe that diet is as effective as drugs in lowering total cholesterol. This again attests to the pervasive message that diets control blood cholesterol, an idea continually reinforced by media advertisements, for example, for the cholesterol lowering effect of breakfast cereal. Whereas it is likely that diet is an important influence on CVD, there is, again, the problem of which diet and the question would probably have been better framed, as in questions 2–4, on specific lipid components. It is likely, for example, that many medical students would not know that dietary cholesterol is largely without effect on serum cholesterol. In any case, it is generally acknowledged that, on average, drugs such as statins have a greater impact on cholesterol than currently reported diet interventions. The general effectiveness of statins and the promotion by pharmaceutical companies has, most recently, led to a movement to reinforce the idea that genetics (which can't be controlled by diet) also plays a role. The competing financial interests have produced, in our view, bizarre and unpatriotic (?) television commercials blaming mother *and *apple pie for high cholesterol.

**Figure 4:**
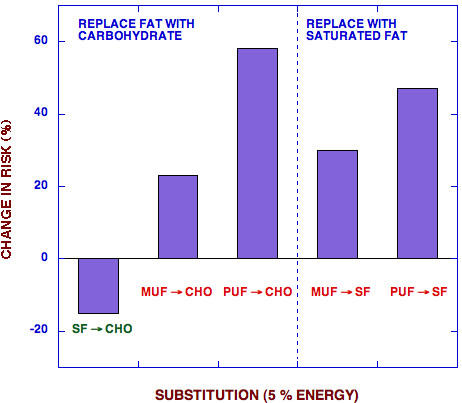
**Effect of substitution of 5 % of calories on incidence of cardiovascular disease. **Data from Hu, *et al*. [8]

Few students in the first year medical class knew that replacing unsaturated fat with carbohydrate was the most damaging substitution in terms of an association with CVD risk. The data from Hu, et al., [8,25] shown in Figure 4 represent yet another reason to reevaluate low fat recommendations. Similar results have been found in the analysis of risk factors [14]. In other words, whereas everybody agrees that removing fat from the diet as a mechanism of calorie reduction is a good thing, replacing fat with carbohydrate correlates with an increase in CVD risk and is likely worse for weight loss.

#### Questions 9–10 – Glycemic Index and Complex Carbohydrates

These two questions (Table 3) point out the confusion that exists in characterizing dietary carbohydrates. The glycemic index (GI), and the glycemic load (GL) which corrects for total carbohydrate in individual foods, are indicators of rise in blood glucose. Glycemic control is a major variable in the analysis of metabolic syndrome and obesity, and dietary strategies based on the glycemic index [28] have the same rationale as low carbohydrate diets: reduce fluctuations in insulin and associated anabolic effects. A low carbohydrate diet might be described as a very low glycemic load diet. Nonetheless, the concepts of GI and GL have become part of the political controversies surrounding dietary strategies and proponents usually urge a low GI diet as an alternative rather than a variation of low carbohydrate diets ([15]) despite the fact that in at least one isocaloric comparison of high GI and low GI meals, the low GI meal was, in fact, lower in carbohydrate [27]. An important limitation on the concept of GI is that fructose and therefore fructose-containing products such as sucrose and high-fructose corn syrup may have low values, although these substances may not be desirable. The atherogenic qualities of fructose [32] is one of the ideas that we bring out in the lectures in the medical school course.

**Table 3:** Student Responses (%) on Carbohydrates

**9. Glycemic Index**	**Low**	**Medium**	**High**	discrim coeff
White Bread	5	22	**70**	0.2
Whole Wheat Bread	39	**49**	10	0.18
Ice Cream	5	**16**	77	-0.1
Carrots	68	**19**	9	0.07
Sucrose	1	10	**86**	0.16
Fructose	**3**	22	73	-0.04
Bran Muffin	25	**49**	**23**	
Banana	20	**53**	23	0.23
				

**10. Complex Carbohydrates**	**Yes**	**No**		
White Bread	60	38		
Whole Wheat Bread	68	32		
Ice Cream	31	68		
Fructose	11	88		
Sucrose	18	81		
Corn Starch	48	51		
Fiber	43	56		

We recommend that the term complex carbohydrates not be used since, in practice, it has lost its original meaning of polysaccharide. It is interesting that, to some extent, student answers followed the original definition. Most students picked both white bread and whole wheat bred as complex although, with a slight preference for picking whole wheat over white bread as many health professionals and the lay public might.

#### Question 11 – Vitamins

We credit the popular media with the generally good knowledge about the antioxidant vitamins shown in Table 4. In our view, however, this may be a mixed blessing because it shifts the emphasis from macronutrient composition, a major factor in health, to micronutrients, which, at least for the American population, has to be considered secondary. The relatively low performance and good discrimination coefficient in the question on redox precursors is somewhat discouraging, especially in that students had been exposed to the involvement of the three oxidative coenzymes in glycolysis and the TCA cycle. Moreover, the origin of NAD coenzymes in dietary niacin was explicitly taught. We think this apparent deficiency likely results from a lack of emphasis on integration of nutritional information with biochemistry.

**Table 4:** Student Responses (%) on vitamin question

**11. Vitamins with indicated activity.**	**yes**	**discrim coeff**
**ANTIOXIDANTS**		
Ascorbic Acid	**77**	0.47
Niacin	23	0.39
Riboflavin	19	0.45
Thiamine	9	0.57
Pyridoxal Phosphate (Vitamin B-6)	21	0.31
Vitamin B-12	31	0.41
Vitamin D	16	0.38
Vitamin E	**69**	0.45
		

**REDOX PRECURSORS**		
Ascorbic Acid	27	0.47
Niacin	**51**	0.31
Riboflavin	**43**	0.23
Thiamine	**34**	0.31
Pyridoxal Phosphate	33	0.28
Vitamin B-12	27	0.45
Vitamin D	11	0.44
Vitamin E	5	0.43

#### Questions 12–14 – Questionnaires as a teaching method: folate metabolism

These questions were presented as a preview for an upcoming lecture on folate metabolism; therefore it was expected that students would not score very highly (Table 5). We identify folate metabolism as one of the critical areas of biochemical nutrition. The importance of homocysteine and use of dihydrofolate reductase inhibitors such as methotrexate are two of the most obvious examples of how biochemistry is a practical part of medicine. At the same time, the biochemical pathways are among the most complex, and because folate spans different areas of metabolism, it is difficult to teach. The key nutritional issues are covered both in lecture and in a case-based learning session.

**Table 5:** Student Responses (%) on folic acid questions

	**Students**	**discrim coeff**
**12. Megaloblastic anemia: deficiencies of**		
Vitamin B-12	39	-0.11
Folic Acid	31	0.17
Neither	6	0.09
**Either**	**19**	**-0.09**
		

**13. Folic acid: relieve deficiencies of**		
Vitamin B-12	7	-0.1
**Folic Acid**	**49**	**0.12**
Both	38	-0.07
Neither	3	
		

**14. Vitamin B-12 deficiency commonly seen in**		
children due to poor nutrition.	32	-0.11
children due to poor absorption.	23	-0.04
the elderly due to poor nutrition.	9	0.1
**the elderly due to poor absorption.**	**25**	**0.1**

### Nutrition in the Medical School

Many papers have been written on the need for, and the difficulty in implementing, improvements in teaching nutrition in medical schools [2,3,33]. Some of the major problems frequently cited are 1) inflexibility in the curriculum due primarily to time constraints and 2) inability to define what aspects of the subject needs to be taught. There is also considerable disagreement on the best method of teaching the subject. The current study bears on some of these questions.

### Adding nutritional material to the curriculum

With respect to point 1) above, the first year medical school curriculum is undoubtedly very dense in content. Adding new material is difficult, especially if it is of the strictly factual type, e.g. macronutrient composition of particular foods. The "low pressure" quiz used here can, in theory, impart a certain amount of specific knowledge and generate student interest without interrupting the general flow of course work. The quiz provides a venue in which interested students can absorb the information, and become aware of the general area if they need to find the information later. Also, in our view, many subjects taught in basic science courses already have nutritional relevance, e.g., cofactors that come from vitamins, and these ideas should be better emphasized. We point out, when the NAD cofactors are introduced, that one would expect global effects of a deficiency disease because of the number of different enzymes that use these cofactors. Although vitamin deficiencies are rare in the absence of gross malnutrition, the emerging role of hypervitamin therapies [34] has great pedagogical value. The tie-in through the quiz may reinforce the basic biochemistry.

### What to teach in nutrition. Guidelines on macronutrient recommendations

We see the question of what to teach as the most critical problem in introducing or expanding nutrition education in the medical school course. Individual faculty may be resistant to giving up their own interests, but this may depend on how well the case is made for changing to new topics.

The original study by Flynn was designed to test physicians' knowledge and expand it to allow them to better implement ATP III recommendations on serum triglycerides. In combination with other questions that we have introduced, the general problem arises as to whether these recommendations or nutritional data should be taught. We feel that official recommendations, such as ATP III, have some inherent contradictions. Given that low fat diets tend to raise triglycerides, the associated recommendations to reduce dietary fat and to raise carbohydrate intake appear somewhat contradictory. The major focus of ATP III, however, is control of cholesterol but again, the literature is not clear-cut. Thus, whereas the association between cholesterol levels and CVD is generally accepted by all but a minority of critics, the effect of diet, especially reduced fat diets, on CVD, or even cholesterol, is far more controversial. The Chapter on "Diet and Coronary Heart Disease (CHD)" in Willett's Nutritional Epidemiology [35] is 40 pages long with more than 300 references and contains more than one disclaimer on the diet-heart hypothesis, e.g. "Even if a change in dietary lipids influences the incidence of CHD in the direction predicted by its effect on total blood cholesterol level, the quantitative relationship between this dietary change and risk of disease is uncertain because of the possibility of many other potential physiologic effects of this dietary manipulation (p. 422), " or "Although substantial indirect evidence supports the classic diet-heart hypothesis, the magnitude of any association is likely to be modest for ranges of diet found within western culture or attainable by realistic dietary changes if the effects predicted by metabolic studies are correct (p. 443)." Finally, papers have been published by respected authors with such titles as: "Dietary fat is not a major determinant of body fat [36]," or "Do high carbohydrate diets prevent the development or attenuate the manifestations (or both) of syndrome X? A viewpoint strongly against [37]" These cautionary reports as well as those of other critics of low fat dietary recommendations [8,19,38-40] are largely ignored by the ATP III and the body of experts who are making current recommendations. The recent demonstration of a beneficial effect of saturated fat and lower carbohydrate in patients on an overall low fat diet [41] has been described in an accompanying editorial as an "American paradox." [42]. The extent to which researchers seek to resolve this paradox remains to be seen.

The analysis above also bears on the role of low carbohydrate diets in educating students and physicians. We have previously indicated how such diets can be used to teach basic intermediary metabolism [43] and whereas we do not recommend any particular diet, we feel that the biochemical rationale of carbohydrate restriction makes it increasingly difficult to justify exclusive recommendations for low fat, high carbohydrate guidelines.

In summary, what to teach remains very problematic. There are clear inconsistencies in the dietary recommendations of the ATP III and other professional agencies. This has to make one question whether students and physicians should be educated only in currently recommended practice, or whether we should instead emphasize understanding the underlying data. This is especially true, given the disclaimers in the American Heart statement [31] that "These recommendations may require modification, based on the results of ongoing and future dietary therapy studies." and that "The available data suggest that it is unlikely that one approach is appropriate for all patients." Of course, presentation of such controversial questions can be introduced into a problem-based learning session but medical students naturally prefer concrete answers and appropriately expect some guidance. The resolution currently depends on individual instructors and departments. It would be good pedagogically to establish the idea that not everything is known about nutrition and that many people consider that a rush to guidelines on insufficient evidence is to be avoided.

## Conclusions

A questionnaire, derived in part from one previously published to assess physician knowledge, can be used to determine medical student awareness of nutritional facts. At the same time, such a quiz can be employed as a teaching device to reinforce earlier material, provide preview of new material, or expose students to factual information that is not easily incorporated into a formal course. One of the areas chosen, the effect of macronutrients on obesity and cardiovascular disease, can lead to discussion and focus on important current issues. The performance of first year medical students as well as the performance of the physicians in the previous study suggest that improvement is needed in imparting knowledge about some basic ideas in nutrition. We believe that the focus should be on these ideas rather than on official recommendations with which the ideas are sometimes in conflict.

Finally, the questionnaire is intended as a practical method. The authors would be grateful for any information on the outcome of its use and/or any suggestions for improving the quiz itself.

## List of Abbreviations

HDL: High Density Lipoprotein

LDL: Low Density Lipoprotein

TAG: Triacylglycerol

THF: Tetrahydrofolic acid

TLC: Therapeutic Lifestyle Changes Diet

ATP III: Third Report of the National Cholesterol Education Program Adult Treatment Program

NCEP: National Cholesterol Education Program

GI: glycemic index

GL: glycemic load

CVD: Cardiovascular Disease
